# *Conidiobolus coronatus* induces oxidative stress and autophagy response in *Galleria mellonella* larvae

**DOI:** 10.1371/journal.pone.0228407

**Published:** 2020-02-03

**Authors:** Michalina Kazek, Agata Kaczmarek, Anna Katarzyna Wrońska, Mieczysława Irena Boguś

**Affiliations:** 1 The Witold Stefański Institute of Parasitology Polish Academy of Sciences, Warsaw, Poland; 2 BIOMIBO, Warsaw, Poland; UT Southwestern Medical Center, UNITED STATES

## Abstract

Cell homeostasis requires the correct levels of reactive oxygen species (ROS) to be maintained as these regulate the proliferation and differentiation of cells, and control the immune response and inflammation. High levels of ROS can cause oxidative stress, leading to protein, lipid and DNA damage, or even cell death. Under physiological conditions, the rate of autophagy remains stable; however, it can be accelerated by a number of exogenous stimuli such as oxidative stress, starvation or hypoxia, leading to cell death. The present paper examines the effect of *Conidiobolus coronatus* infection on the immune response, oxidative stress processes and autophagy in the greater wax moth, *Galleria mellonella*. Fungal infection was found to result in the disorganization of the cytoskeleton of the larval immune cells and the enhancement of oxidative defense processes. Lipid peroxidation and autophagy were also induced in the hemocytes. Our findings show that *G*. *mellonella* is an ideal model for exploring immune mechanisms.

## Introduction

Insects rely on multiple immune responses that employ both humoral and cellular defense reactions. Typical cellular responses include the phagocytosis of small pathogens such as bacteria and fungi, the encapsulation of parasitoid wasps, nematodes and other larger parasites, or nodulation by specific immune cells known as hemocytes [1,2]. It has been suggested that five classes of hemocytes are present in the Lepidoptera: prohemocytes, plasmatocytes, granulocytes, spherulocytes and oenocytoids. Humoral reactions include the production of antimicrobial peptides (AMPs), reactive oxygen and nitrogen species, as well as the use of the prophenoloxidase (proPO) activating system, which regulates the coagulation and melanization of hemolymph [3,4].

**Fig 1:**
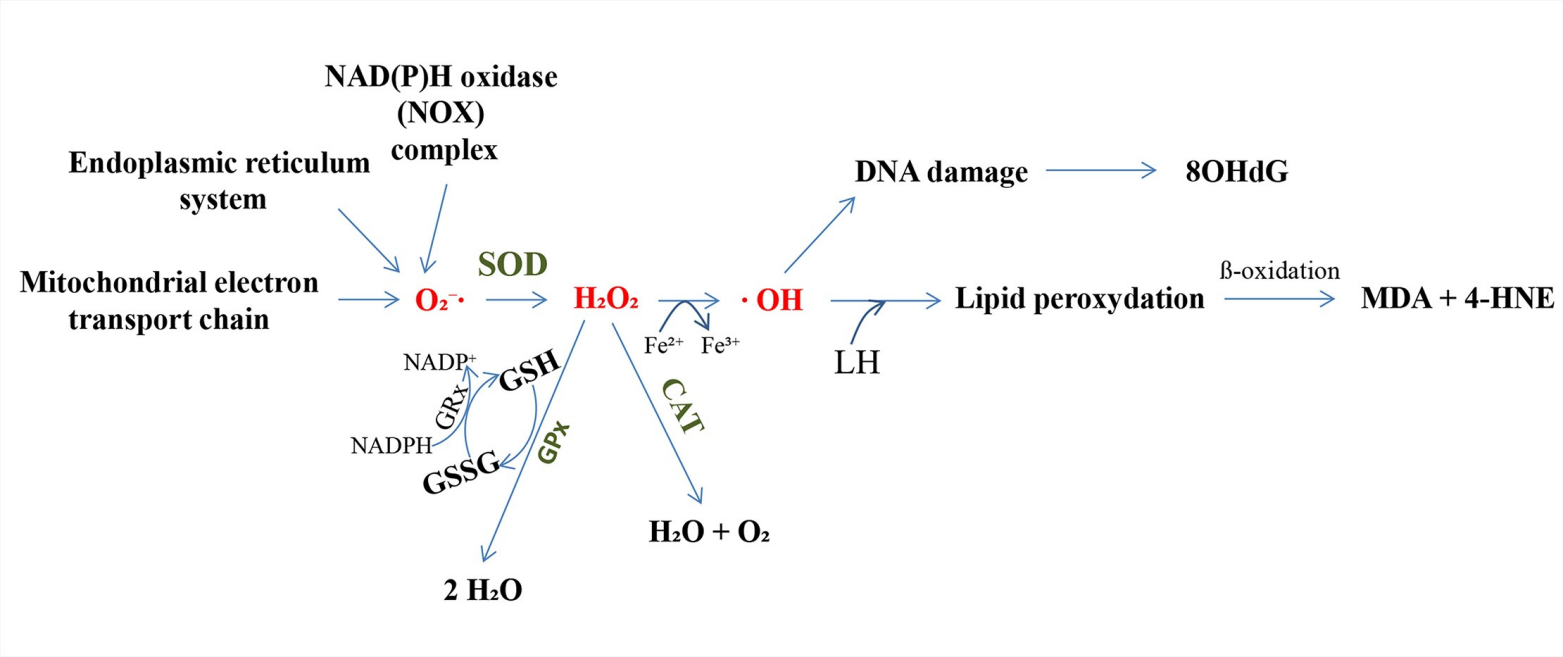
The general scheme of action of reactive oxygen species (ROS) and the antioxidant defense system. Main sources of ROS generation include the mitochondrial electron transport chain, endoplasmic reticulum system, and NAD(P)H oxidase (NOX) complex. The oxygen utilized for respiratory purposes can be converted to ROS such as O_2_⁻∙, H_2_O_2_, and ∙OH. Three key enzymes forming the defensive complex against ROS are SOD, CAT and GPx. Symbols used: GPx–glutathione peroxidase; GRx–glutathione reductase; GSSG–oxidized glutathione; GSH–reduced glutathione; H_2_O_2_ –hydrogen peroxide; NADPH–reduced nicotinamide adenine dinucleotide phosphate; SOD–superoxide dismutase.

Reactive oxygen species (ROS) such as singlet oxygen, ·OH radicals and H_2_O_2_, play a dual role in living organisms: although they are needed for the regulation of repair processes, metabolism and gene expression, they are also responsible for lipid peroxidation, protein carbonylation and DNA oxidation, and can reduce the availability of glutathione [5–7]. Mitochondria are the main source of reactive oxygen species in eukaryotic cells. Under physiological conditions, approximately 95% of oxygen is reduced to water molecules during its passage through the mitochondrial electron transport chain in the presence of cytochrome oxidase, while the remaining 5% is converted to oxygen radicals. It is possible for ROS concentrations to exceed specific values inside the cells, resulting in the phenomenon known as oxidative stress and leading to the development of many radical-related diseases [8,9]. However, many enzymatic and non-enzymatic defense mechanisms serve to efficiently convert reactive oxygen species to less reactive substances (Fig 1) [10].

Insects express a large number of antioxidant enzymes, including superoxide dismutase (SOD), catalase (CAT), ascorbate peroxidase (APOX), glutathione peroxidase (GPx) and glutathione S-transferases (GSTs) [11]. Three of these, *viz*. SOD, CAT and GPx, form the main defensive complex against ROS. SOD catalyzes the dismutation of superoxide radicals to H_2_O_2_ and molecular oxygen; following this, H_2_O_2_ is scavenged by CAT, resulting in the production of water and molecular oxygen [12]. Finally, H_2_O_2_ is reduced to water by GPx. As GPx is believed to be present at only very low levels in insects, its activity is compensated for by CAT and glutathione S-transferase with peroxidase-like (GSTpx) activity [13]. The GSTs constitute a group of multifunctional detoxification enzymes present in both invertebrates and vertebrates [14].

The antioxidant capacity of insects also employs non-enzymatic ROS scavengers such as glutathione and ascorbate. The most important property of glutathione is its antioxidative action, but also it takes part in the remodeling of damaged components of cells, mostly the lipids and proteins of cell membranes. Furthermore, through its participation in maintaining the proper redox potential in the cell, glutathione supports the regulation of the intracellular metabolism, and participates in cell growth, cell differentiation and apoptosis [15,16]. It is also known to act as a neuromodulator and neurotransmitter. Interestingly, neurodegenerative disorders such as Parkinson's disease and Alzheimer's disease, as well as diabetes or immunodeficiency are associated with lower levels of glutathione, as are conditions of strong oxidative and nitric stress. In eukaryotic cells, the reduced form of glutathione (GSH) predominates, with the oxidized form (GSSG) constituting only a small percentage of the total glutathione level.

The oxidation of glutathione to the disulfide form can occur via an enzymatic pathway, based on glutathione peroxidase and reductase, and a non-enzymatic pathway. Glutathione peroxidase (GPx) is the first selenoenzyme to be identified (84 kDa). It is mainly present in the cytosol, but is also found in lower quantities in mitochondria and the nucleus. GPx acts as a catalyst for the reduction of hydrogen peroxide by GSH, and its main function is to protect cells from oxidative stress by preventing peroxidative damage to cell membranes [17]. Reducing the glutatione level results in lower GPx activity.

Glutathione reductase (GRx) is another enzyme involved in the regulation of glutathione (22 kDa) [18]. GRx is present in the cytosol and mitochondria. It is responsible for maintaining the appropriate level of GSH by rebuilding the reduced form of glutathione at the expense of NADPH oxidation [18,19]. When the level of glutathione is significantly lowered, the oxidized form of glutathione is increased, leading to the inhibition of glutathione peroxidase and saturation of the reductase enzyme.

One of the most predominately examined processes connected with ROS and their effects are lipid peroxidation. Lipid peroxidation, the oxidation process of unsaturated free fatty acids [8,9], is well known to induce cellular injury and is used as an indicator of oxidative stress. Lipid peroxides are unstable and decompose to form a complex series of compounds through a process consisting of three main steps: initiation, propagation and termination [8]. Peroxidation processes are particularly destructive in cells exposed to oxidative stress, such as during infections and inflammation, and during neurodegenerative diseases and cancer. In all cases, the process results in damage and depolarization to the cytoplasmic membranes and increased production of harmful free radicals in cells.

Polyunsaturated fatty acid peroxides generate malondialdehyde (MDA) and 4-hydroxyalkenals (HAE) upon decomposition. These two compounds can damage nucleic acid and protein molecules [8,20,21]. As a result, numerous other aldehydes, hydroxyaldehydes (2-aldehydes, hepta-2,4-dienal) and hydrocarbons (ethane, pentane) are also formed. Lipid peroxidation products alter the physical properties of cell membranes, ultimately resulting in lowered integrity. MDA and HAE levels are often used as indicators of lipid peroxidation, and MDA is commonly used as a marker of oxidative stress.

There is comprehensive experimental evidence that oxidative damage can influence not only the lipids of cellular membranes, but also proteins and DNA. Two of the most commonly-used markers of oxidatively-modified DNA molecules are 8-hydroxy-2-deoxyguanosine (8-OHdG) and/or 8-oxo-7,8-dihydro-2-deoxyguanosine (8-oxodG). Over recent years, 8-OHdG has been widely used in many studies as a biomarker for the measurement of DNA oxidative damage, as well as a risk factor for many diseases, including cancer.

Autophagy is a nonselective process of bulk cytoplasmic degradation [5–7] consisting of the sequestration of the cytosol, the fusion of primary lysosomes, and the subsequent degradation of proteins in autophagosomes. The process is evolutionarily conserved among eukaryotic organisms ranging from yeasts to mammals [22,23]. It is also believed that autophagy plays an essential role in maintaining cellular homeostasis and is involved in the pathogenesis of numerous diseases [24]. A wide range of signal pathways are based around the interaction between ROS level and autophagy in response to stress conditions, as the two interact to maintain homeostasis on the cellular level: ROS can induce autophagy, but autophagy can also serve to control the level of ROS in cells and decrease their toxic effects [22] [25]. ROS regulates autophagy via several signaling pathways such as the AMPK-mTOR and PI3K-Akt-mTOR pathways [26]. Additionally the presence of high levels of ROS can lead to a redox imbalance via the release of GSH into the environment, thus decreasing the GSH/GSSG ratio and potentially inducing autophagy [27].

Autophagy is sub-divided into three pathways: chaperone mediated autophagy (CMA), macroautophagy and microautophagy. CMA has only so far been described in mammals, and is believed to be involved in the degradation of single, soluble proteins. In contrast, the activities of macro- and microautophagy are highly dependent on the organism, the inducer of autophagy and environmental conditions [7], and the two processes may occur at the same time within the same organism [28]. For example, during macroautophagy, a double membrane vesicle called the autophagosome forms around the cytosolic components, which subsequently fuse with the lysosomes and degrade their contents [22]; however, during microautophagy, the components to be degraded are sequestrated by the vacuolar membrane without prior sequestration in the autophagosomes. In addition, in mammals, two ubiquitylation-like modifications, Atg12-conjugation and LC3-modification, are required during autophagosome formation.

The regulation of autophagy is partly based on the phosphorylation and dephosphorylation of ATG proteins. Recently, 31 Atg genes were identified in yeast, including the two ubiquitin-like systems Atg8 and Atg12 [23,29]. Many mammalian homologs of yeast Atg genes have been identified and characterized, as well as homologs of three Atg8 genes: *LC3*, *GABARAP* and *GATE-16* [30].

Little is known of the autophagy process in insects, and even less about the relationship between autophagy and oxidative stress, especially after fungal infections. A fuller understanding of the interactions between entomopathogenic fungi and insects may allow more efficient use of fungal bioinsecticides in the near future, and more detailed knowledge of the action of fungi and their influence on programmed cell death is needed to better understand fungal-induced pathogenesis in insects. Our findings not only provide important insights into the field of insect physiology but also represent a useful reference for future studies.

## Results

### Infection-induced changes in the insect cytoskeleton

Our previous research showed that following fungal infection, *G*. *mellonella* larvae became immobilized, lost the ability to construct cocoons and ceased silk spinning, which is continuously produced by normal caterpillars [31]. Moreover, upon the termination of exposure to the fungal colony, black spots were observed on the cuticle of larvae that had been in contact with *C*. *coronatus*: these being sites of fungal invasion. A few hours later, the spots spread and joined with neighboring spots. Finally, approximately 48 hours after infection, the larvae were dead and the cuticle of the dying insects had become completely black [31,32].

**Fig 2:**
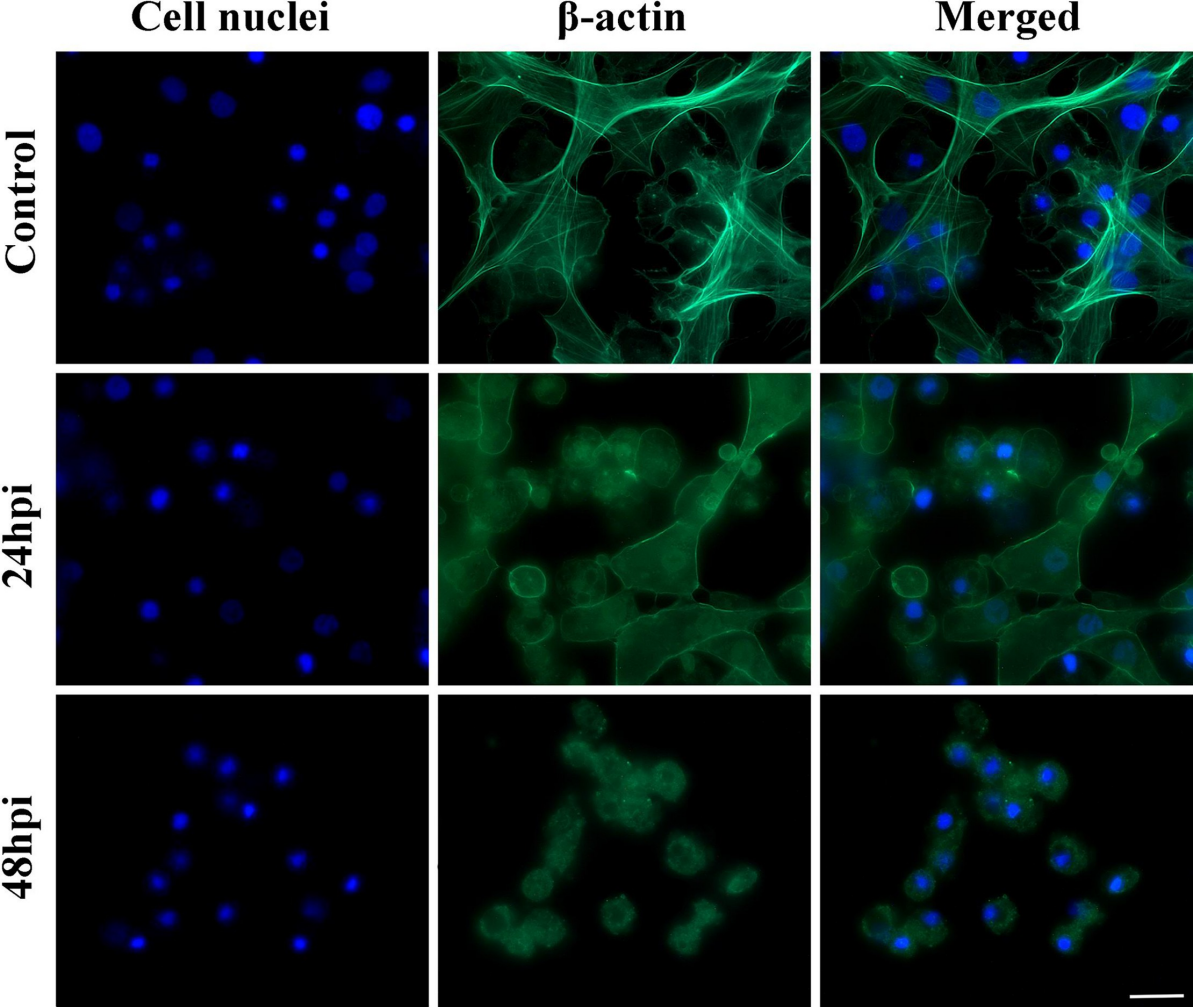
Hemocytes of *Galleria mellonella* larvae after 24-hour *in vitro* culture: (A) control, (B) 24 hpi group, (C) 48 hpi group. Larvae were infected with the entomopathogenic fungus *Conidiobolus coronatus*. Blue color–cell nuclei labelled by DAPI (Ex/Em: 345/455 nm); Green color–actin fibers labelled with ActinGreen 488 kit reagent (Ex/Em: 495/518 nm). Scale bar 20 μm.

In the first part of the experiment, it was found that *C*. *coronatus* infection resulted in damage to the hemocytes of the cytoskeleton, more specifically the actin fibers (Fig 2). It is also worth mentioning that only adherent cells were visible in the 24-hour *in vitro* cell culture, i.e. granulocytes and plasmatocytes: non-adherent types, such as sferulocytes and oenocytoides, were washed out during the staining procedures. As soon as 24 hours after infection, changes in the shape of the cytoskeleton were visible: the cells were more rounded, and lacked a characteristic network for hemocytes. In addition, 48 hours after infection, adherent hemocytes were destroyed, and degranulated granulocytes and vacuolized plasmatocytes were observed that formed microaggregations. Unlike in the control group, no hemocyte fiber network was observed (Fig 2). The actin cytoskeleton of the hemocytes from the infected larvae was disorganized.

### Infection-activated oxidative processes in *Galleria mellonella*

**Fig 3:**
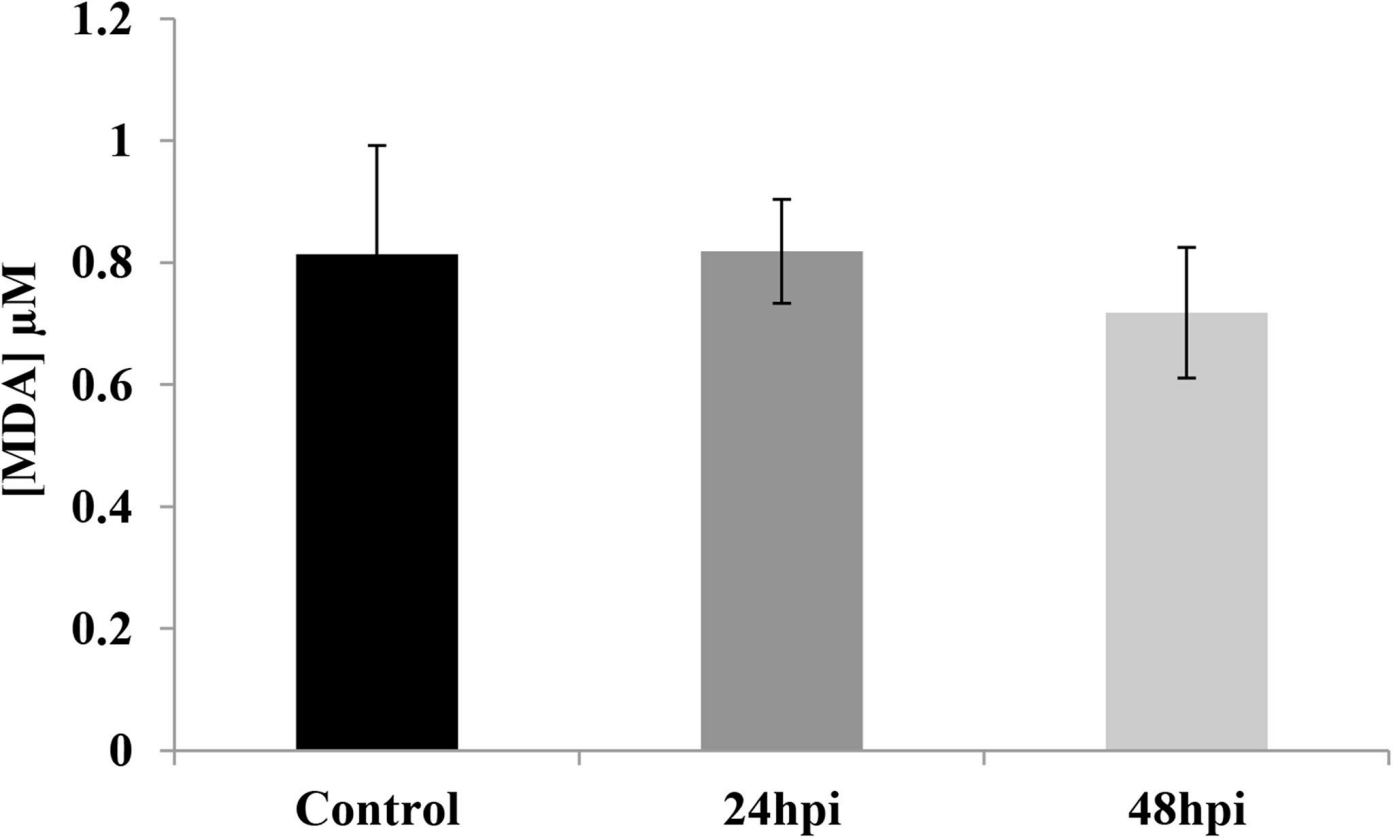
Determination of the level of malondialdehyde (MDA) in hemolymph of *Galleria mellonella* larvae.

**Fig 4:**
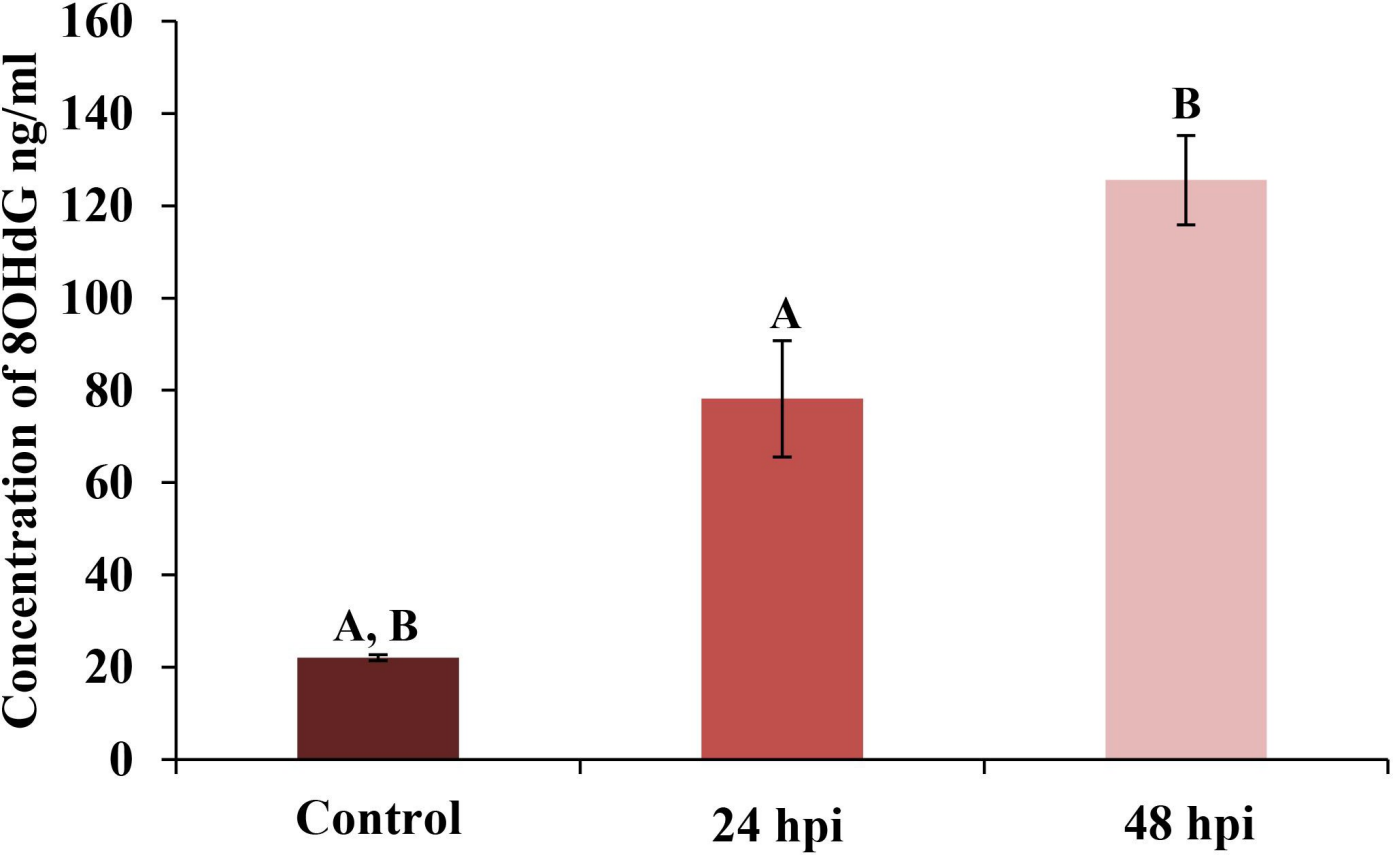
Determination the level of 8-hydroxy-2’-deoxyguanosine (8-OHdG) in hemolymph of *Galleria mellonella* larvae. Statistically significant differences are marked with the same letters (p<0.05).

The levels of MDA and 8-OHdG in the hemolymph are given in Fig 3 and Fig 4, with the data regarding lipid peroxidation in the hemolymph of *G*. *mellonella* after fungal infection given in Fig 3. Very similar MDA levels were observed in the hemolymph of the controls and the 24 hpi group: 0.81 ± 0.18 μM of MDA in the former and 0.82 ± 0.09 μM in the latter. Lower levels were observed in the 48 hpi group, i.e. those exposed to the fungus for 24 hours and incubated for another 24 hours (), but the difference was not significant (0.72 ± 0.11 μM).

The effect of *C*. *coronatus* infection on 8-OHdG content is presented in Fig 4. The 8-OHdG content was found to be 22.05 ± 0.65 ng/ml in the control larvae; however, significantly higher values were observed in the 24 hpi group (78.16 ± 12.85 ng/ml) and the 48 hpi group (125.55 ± 9.64 ng/ml).

### Infection-activated oxidative defense processes in *Galleria mellonella*

**Fig 5:**
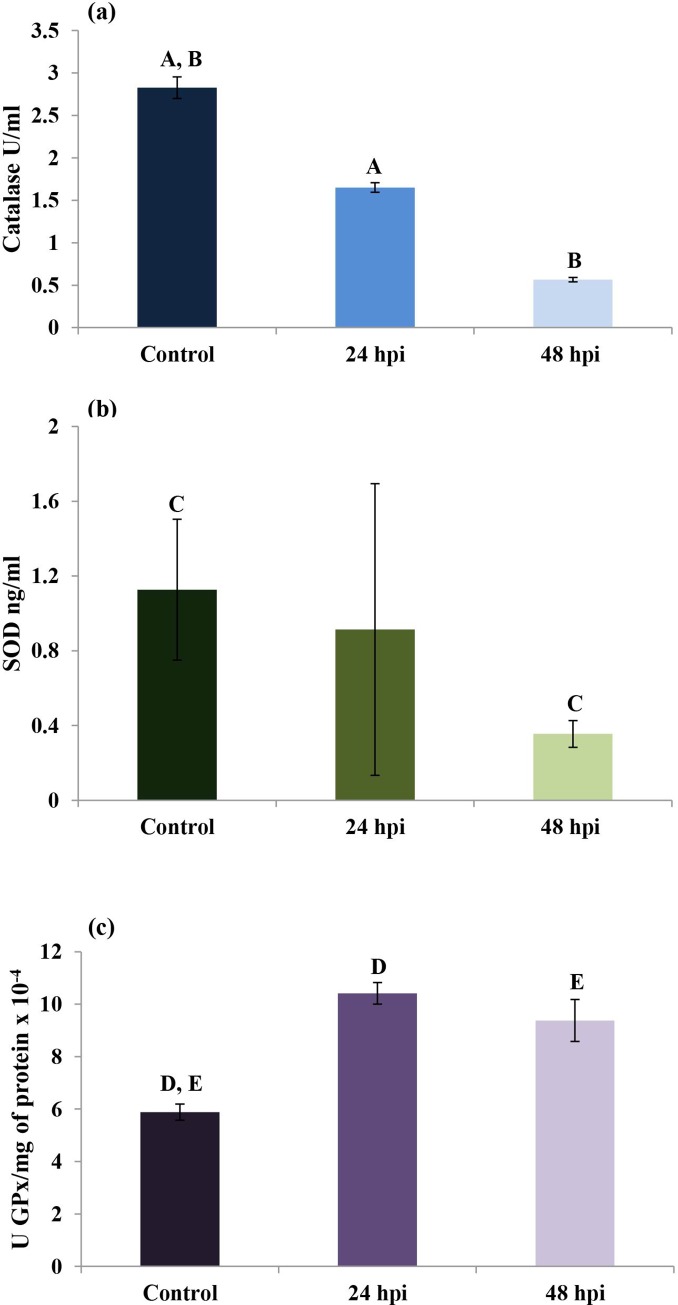
Effect of fungal infection on CAT (a), SOD (b) and GPx (c) activities in the hemolymph of *Galleria mellonella* larvae. Statistically significant differences are marked with the same letters (p<0.05).

The effects of fungal infection on catalase (CAT) (Fig 5A), SOD (Fig 5B) and GPx (Fig 5C) activities in the hemolymph of wax moth larvae were also examined. CAT levels were 2.83 ± 0.13 U/ml in the control group; however, this value was significantly lower in the 24 hpi group (1.65 ± 0.06 U/ml) and the 48 hpi group (0.57 ± 0.03 U/ml). SOD activity was also significantly lower in the 48 hpi group (0.36±0.07 ng/ml) than the control group (1.13 ± 0.38 ng/ml). GPx activity (Fig 5C), recalculated per protein concentration, was significantly higher in the 24 hpi (10.41±0.41 U/mgx10^-4^) and the 48 hpi (9.38±0.80 U/mgx10^-4^) groups than in controls (5.88±0.31 U/mgx10^-4^).

### Induction of autophagy in insect hemocytes

**Fig 6:**
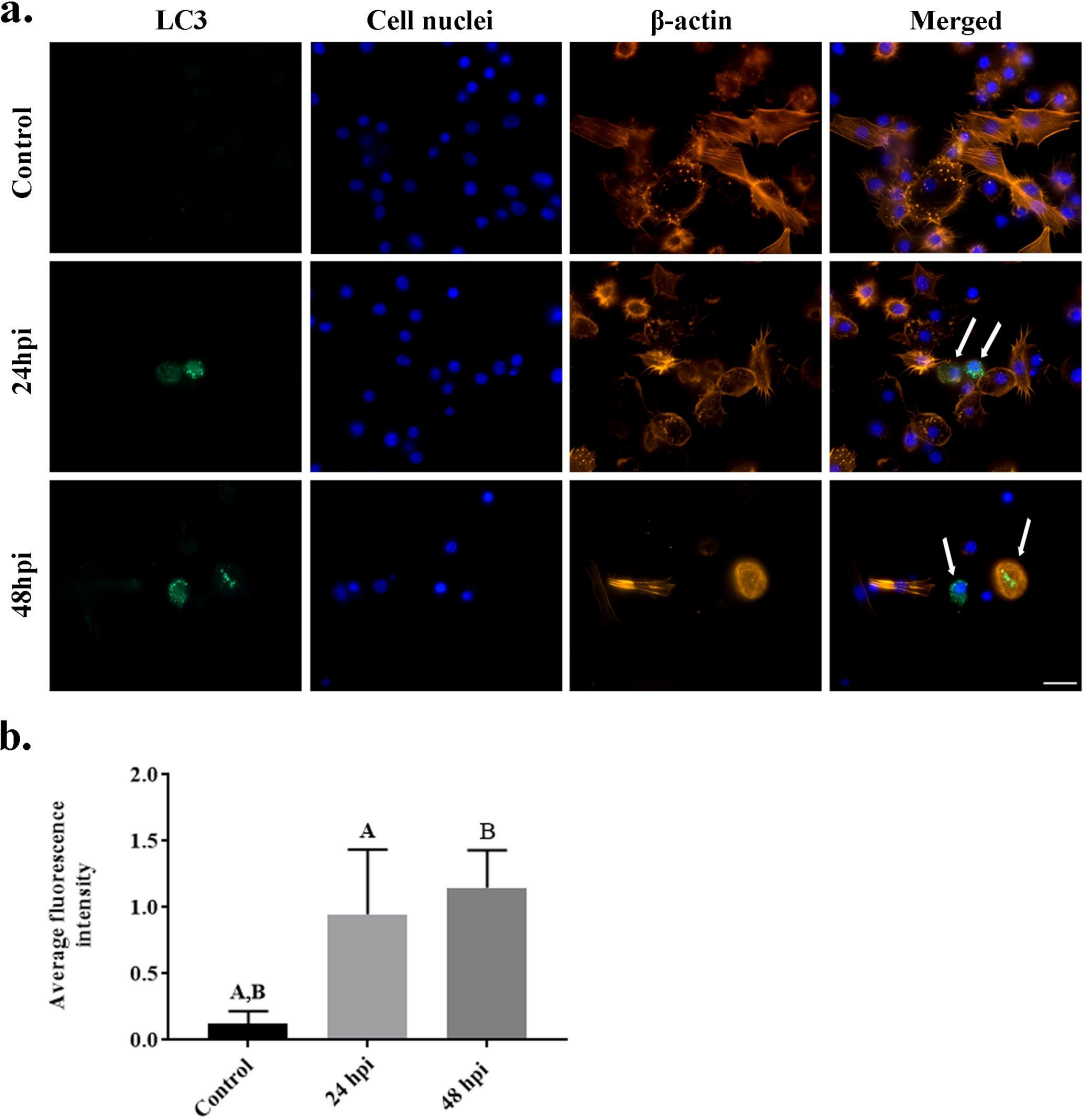
Hemocytes of *Galleria mellonella* control larvae, 24 hours post infection and 48 hours post infection with the entomopathogenic fungus *Conidiobolus coronatus*. (a) The intensity of autophagosomes was evaluated by CYTO-ID® Autophagy Detection Kit staining and (b) their average fluorescence intensity was quantified. Blue color–nuclei labelled by DAPI (Ex/Em: 345/455 nm); Green color– 488nm-excitable green fluorescent detection reagent CYTO-ID® Autophagy Detection Kit (Ex/Em: 495/518 nm). Scale bar 20 μm.

The final part of the experiment examined the influence of fungal infection on the formation of autophagosomes in hemocytes (Fig 6). Hemocytes were stained with an CYTO-ID® Autophagy detection kit and analyzed with fluorescent microscopy (Fig 6A), also the fluorescence intensity was calculated (Fig 6B). The cells in the control group were not stained; however, greater autophagosome staining was observed in the 24 hpi group and much greater staining was observed in 48 hpi group.

**Fig 7:**
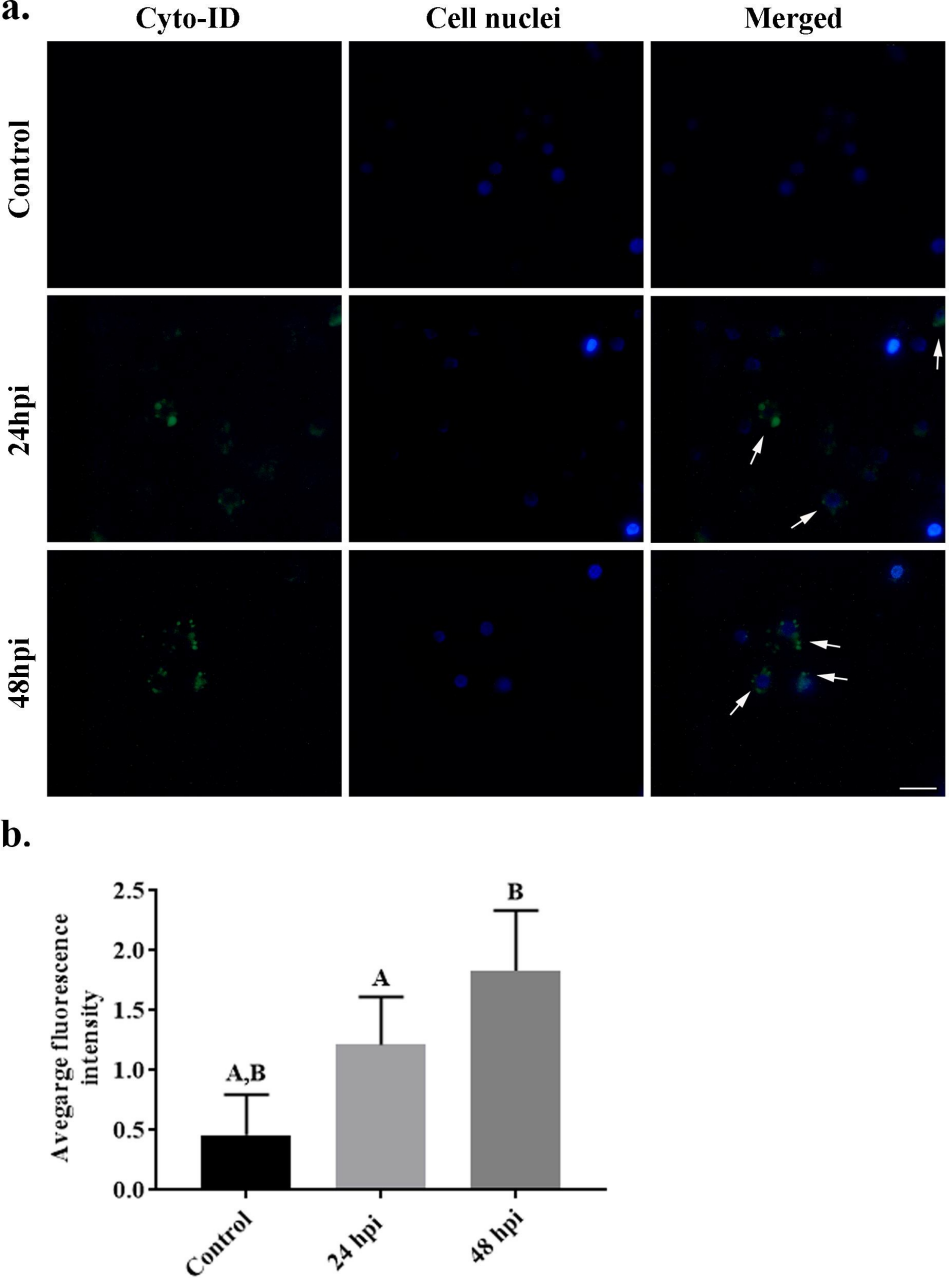
Hemocytes of *Galleria mellonella* control larvae, 24 hours post infection and 48 hours post infection with the entomopathogenic fungus *Conidiobolus coronatus*. (a) The intensity of LC3 protein was evaluated by LC3 antibody staining and (b) their average fluorescence intensity was quantified. Blue color–nuclei labelled by DAPI (Ex/Em: 345/455 nm); Red/orange color–actin fibers labelled with ActinRed 555 kit reagent (Ex/Em: 540/565 nm); Green color–LC3 protein. Scale bar 10 μm.

*C*. *coronatus* infection resulted in the damage of cytoskeleton (actin fibers) of wax moth larvae (Fig 2 and Fig 7) and changes in the shape of the cytoskeleton were clearly visible at 24 hpi. As the results indicated that autophagy could be the main type of cell death in *G*. *mellonella* larvae after fungal infection, the next stage of the study examined the effect of infection on LC3, the main autophagy protein by staining with LC3 antibody (Fig 7). High levels of LC3-positive staining were observed in cells at 48 hours post infection (hpi). Fewer stained cells were detected 24 hpi. No LC3 stained cells were observed in the control groups. The average fluorescence intensities in the examined groups increased to 0.95 for 48 hpi and 1.15 for 24 hpi; these were significantly different to the control value of 0.13 (Fig 7B).

**Fig 8:**
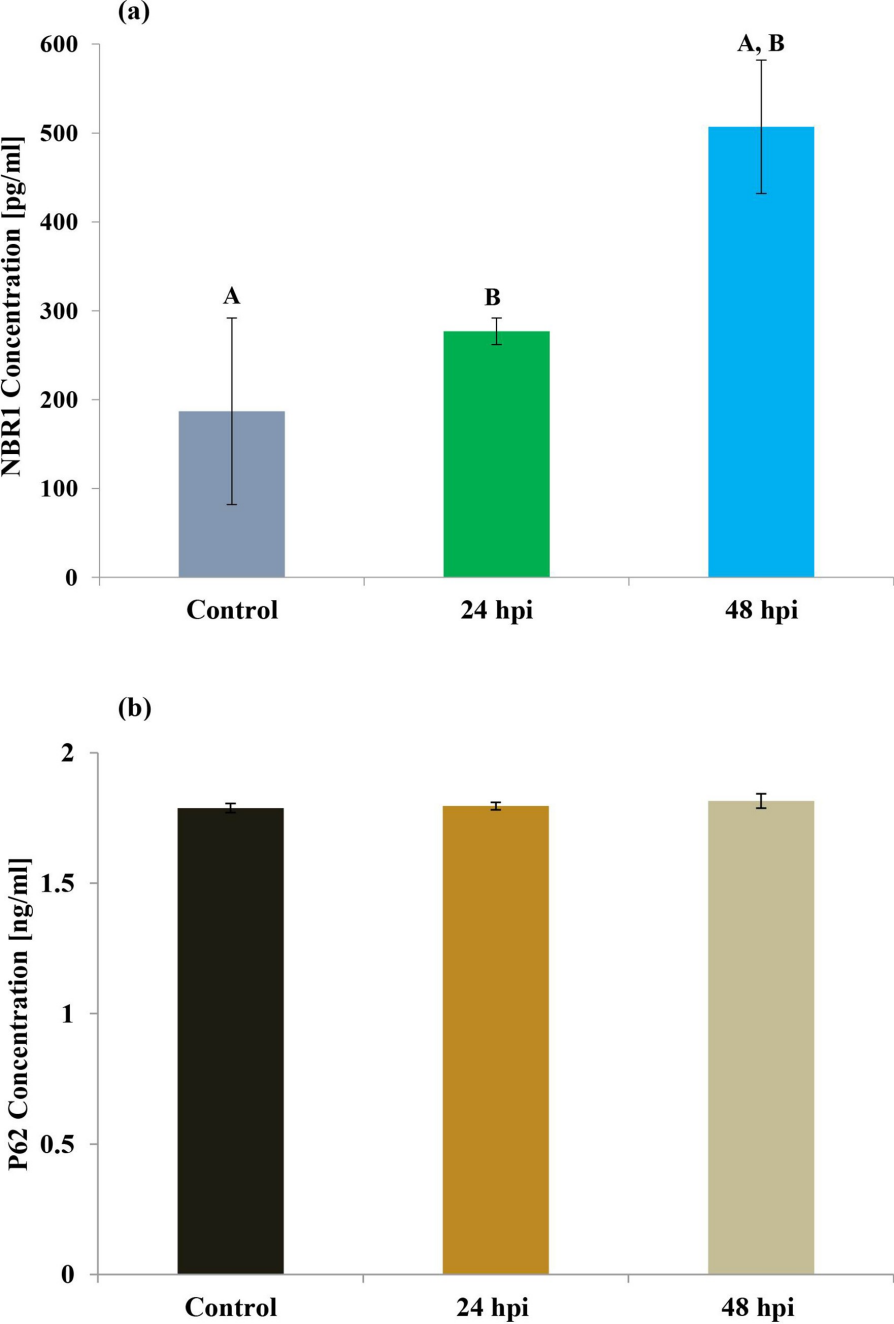
Determination of the level of NBR 1 and p62 protein in hemolymph of *Galleria mellonella* larvae.

The levels of NBR1 and p62 proteins, which are often used as biomarkers to study autophagic flux, were also examined (Fig 8). Although no differences in the level of p62 protein (Fig 8B) were observed between control and infected groups, statistically differences were found in the level of NBR1 protein: its concentration peaked in the 48 hpi culture (507 pg/ml; Fig 8A).

## Discussion

Naturally-occurring entomopathogens, such as fungi or nematodes, are important regulatory factors among insect populations. At present, several species of entomopathogenic fungi are employed as biological control agents of insect pests [33,34]: species of Hirsutella, Metarhizium, Paecilomyces and Beauveria all demonstrate high efficiency against arthropod invasion. Among the Entomophthorales, the members of the genus Conidiobolus are believed to be evolutionarily primitive, with rather narrow host ranges. One such species, *C*. *coronatus*, is a soil fungus which demonstrates entomopathogenicity towards a number of insects, including *G*. *mellonella*, *Dendrolimus pini* [35] and various collembolans [36]. It is known to produce various mycotoxins [37,38], including a 30 kDa protein believed to be toxic to *G*. *mellonella* larvae, coronatin-1 (36 kDa) and coronatin-2 (14.5 kDa) which compromise the function of hemocytes, and the 30–32 kDa serine protease, whose properties resemble subtilisin [35,37–39]. A previous study also has shown that while exposure did not influence the fat bodies, characteristic changes were observed in the Malpighian tubules in *G*. *mellonella* larvae after infection [40].

The remarkable evolutionary success of insects is partly due to their ability to build up a sophisticated, effective, and highly-adaptable defense system against numerous microorganisms, including pathogenic fungi. Insects rely on multiple immune responses that include both humoral and cellular defense reactions. Humoral reactions include the production of antimicrobial peptides (AMPs), reactive oxygen and nitrogen species, and the prophenoloxidase (proPO) activating system, which regulates the coagulation and melanization of hemolymph. Cellular responses include the phagocytosis of small pathogens such as bacteria and fungi, the encapsulation of parasites such as parasitoids and nematodes, and nodulation by specific immune cells (hemocytes) [1,2]. Similarly to Wieloch et al (2011), our present microscopic examination of *G*. *mellonella* hemolymph confirmed the presence of all five classes of hemocytes believed to be present in the Lepidoptera: prohemocytes, plasmatocytes, granulocytes, spherulocytes and oenocytes.

The cytoskeleton is a system of filaments or fibers present in the cytoplasm of all eukaryotic cells, including insect immune cells, and one that serves a wide range of functions. It spatially organizes the contents of the cell, maintains its shape and connects the cell with the external environment. The cytoskeleton is also responsible for cell locomotion. The cytoskeleton consists of three main polymers, actin filaments (microfilaments), microtubules (made of tubulin protein) and intermediate filaments [41]. In the present study, staining with Alexa Fluor 488 dye revealed that *C*. *coronatus* infection had resulted in damage to the actin filaments of the hemocytes of the infected wax moth larvae (Fig 2). The characteristic fiber network for granulocytes and plasmatocytes had disappeared, and 48 hours after infection, these cells had disintegrated. Our results confirm previous findings that pathogenic fungi disrupt the cytoskeleton of insect immune cells [41–43]. Similarly, membrane blebbing, cytoplasm vacuolization and cell swelling has been reported in *G*. *mellonella* hemocytes 18 hours after *Pseudomonas aeruginosa* infection [44]. In addition, swollen hemocytes, disintegrated and membrane blebbing were observed after treatment with coronatin-1, a toxin produced by *C*. *coronatus* [38]. Also, injection of *Metarhizium anisopliae* blastospores into the body of *G*. *mellonella* larvae resulted also in morphological changes in the hemocytes during infection; plasmatocytes isolated from the infected larvae were less able to attach and spread on a glass surface than those from an untreated control group [45].

The present study examined the effect of *C*. *coronatus* on *G*. *mellonella* immune cells, particularly the morphology of larval hemocytes, which play a crucial role in the defense against numerous pathogens. Our findings indicate that wax moth larvae activate immune mechanisms and initiate programmed cell processes in response to entomopathogenic fungal infection. Previous studies on the immune cell response of wax moth larvae during infection with *C*. *coronatus* have focused exclusively on apoptosis. Similarly, our own previous work has examined the activity of caspases 1, 2, 3, 5, 8 and 9 using commercially-available kits (Enzo Life Sciences). Caspases are crucial mediators for apoptosis which can be subdivided into two groups: initiator caspases (i.e. caspase-2, -8, -9 and -10) and effector caspases (i.e. caspase-3, -6 and -7). Statistically-significant results (p≤0.05) were obtained only for caspase 8 and 9 [46]. While caspase-8 is activated during the extrinsic pathway, which is dependent on membrane receptors known as death ligands, caspase-9, associated with mitochondrial changes, is activated by the intrinsic pathway. In contrast, caspase-3 is activated in the apoptotic cell by both the extrinsic (death ligand) and intrinsic (mitochondrial) pathways. As it is responsible for the majority of proteolysis during apoptosis, the presence of cleaved caspase-3 is considered a reliable marker for cells that are dying, or have died, by apoptosis. In addition, caspase-3 is a caspase protein which interacts with caspase-8 and caspase-9 [47,48]. However, as previous studies have found no significant differences in the activity of caspase-3 between control and examined insects [46], the present study focuses on the second type of programmed cell death: autophagy.

Autophagy is a well-understood process which has been extensively described in both *Saccharomyces cerevisiae* and mammal cells. Recently, 31 Atg genes were identified in yeast, including the two ubiquitin-like systems Atg8 and Atg12 [23,29]. Many mammalian homologues of yeast Atg genes have also been identified and characterized. In mammals, homologues for three Atg8 genes, *LC3*, *GABARAP* and *GATE-16*, have been identified and well characterized [30]. Despite this, our knowledge of autophagy in insects remains incomplete. The most comprehensively studied insect in this regard is *Drosophila melanogaster*: in this case, autophagy, not apoptosis, is believed to be essential for programmed death cell in the midgut [49], and starvation rapidly induces autophagy in the fat bodies of the larvae [6]. Under certain conditions in Drosophila, midgut and salivary gland tissues show both high caspase activity and autophagosome formation [50,51], suggesting that apoptosis and autophagy may be highly integrated. In addition, mosquitoes are known to activate autophagy following vitellogenesis to recover excess yolk protein [52,53]. In Lepidopteran species, a chain of events including apoptosis and autophagy has been observed during massive remodeling and elimination of tissue during metamorphosis [23,54]. The process has also been associated with the Atg8 protein GmAtg8 of the wax moth, *G*. *mellonella*, where it is expressed in the midgut, ovary, Malpighian tubules, fat body and silk gland. Our present findings confirm the presence of LC3-positive hemocytes in *G*. *mellonella* 24 and 48 hours after fungal infection (Fig 7), as well as autophagosome formation characteristic of autophagy (Fig 6).

Substrate selection during autophagy is mediated by ubiquitylation and the recruitment of ubiquitin-binding autophagic receptors such as p62 and NBR1 [55,56]. It has been shown that these receptors act cooperatively to target some types of substrates in emerging autophagosomes; NBR1 is also known to play an essential role in the selective autophagic degradation of peroxisomes (pexophagy), with its presence being sufficient for degradation. NBR1 is also an LC3- and Ub-binding protein; although NBR1 is degraded by autophagy depending on its LC3-interacting region (LIR), p62 is not strictly required for this process. In the present study, the level of NBR1 protein was found to be elevated in wax moth hemolymph after fungal invasion, while p62 protein level remained stable, even after infection (Fig 8); it is possible that autophagy took place without the need for p62 protein. Our results confirm those of previous studies showing that hemocytes of wax moth induce the autophagic pathway [44,57]. Further evidence that these two processes might interact and coincide with each other was given by Mizerska-Dudka and Andrejko (2014), who observed features typical for both apoptosis and autophagy in wax moth hemocytes following *Pseudomonas aeruginosa* infection. However, it remains unclear whether wax moth hemocytes are targeted to apoptotic or autophagy cell death after *C*. *coronarus* infection, and additional tests are required. It is possible that wax moth larvae may demonstrate autophagy as a response to fungal infection, a high-stress situation, to maintain physiological homeostasis, and that this process can be activated after ineffective apoptosis induction; alternatively, both autophagy and apoptosis may interact and be observed on different levels.

Finally, our results suggest that after exposure to the entomopathogenic fungus, the circulating hemocytes ate funneled into the autophagic pathway under the influence of by stress and reactive oxygen species (ROS). Connections between ROS and autophagy have been observed during diverse pathological conditions, but the mode of action and its purpose remain poorly understood [58,59]. Our present findings do not indicate any differences in the level of MDA in the hemolymph of *G*. *mellonela* after fungal infection (Fig 3); however, the level of 8-OxoG was elevated (Fig 4). These results suggest that after entomopathogenic fungus infection, larval hemocytes are subject to DNA damage rather than lipid peroxidation. Lipid peroxidation and its products, such as malondialdehyde, have been observed in insects after exposure to entomopathogenic fungi. Chaurasia et al.(2016) [60] report an increase in MDA level in the fat bodies and gut of *Periplaneta americana* after exposure to *M*. *anisoploae*, *Isaria fumosoroseus* and *Hirsutella thompsonaii*, while its level decreased in the hemolymph and the rest of the body. Similar results were obtained by [61], who note a lower level of MDA in cockroaches injected with *M*. *anisopliae* spores. Currently, nothing is known about the response associated with 8-OxoG in insects following infection.

As mentioned above, insects have evolved a range of mechanisms based on enzymatic (SOD, CAT, GPx) and non-enzymatic antioxidant systems to mitigate the effects of oxidative damage. Under normal physiological conditions, all the antioxidant enzymes interact with each other. In the present study, the level of GPx was found to be elevated in the hemolymph after *C*. *coronatus* infection, while those of CAT and SOD were lowered (Fig 5). GPx plays a key role in the management of lipid peroxidation, and any dysfunction in this enzyme results in the accumulation of lipid peroxides and potentially, cell death [8]. This may suggest that *G*. *mellonella* antioxidant enzymes are effective against the harmful effects of lipid peroxidation. In addition, we also observed an accumulation of 8-OxoG, a DNA damage biomarker: it is reasonable to assume that infection had induced a high level of DNA damage and that the insect hemocytes were subject to autophagy.

To summarize, it appears that *G*. *mellonella* hemocytes use the glutathione repair system to prevent oxidative stress during fungal invasion. No lipid peroxidation was observed following infection, but a high degree of DNA damage was implied by the elevated level of 8-OxoG. As a result of these processes, hemocytes enter the autophagic pathway, indicated by the presence of LC3 and NBR1 proteins. However, further studies are needed to obtain a deeper insight into this very interesting phenomenon.

We present our preliminary results concerning *G*. *mellonella* larvae cellular immune response after entomopathogenic *C*. *coronatus* infection. Our findings provide information about the cellular immune response in *G*. *mellonella* larvae and the connection between this process and those associated with oxidative stress and autophagy; this data can improve our understanding of the host-pathogen interaction. Such an understanding of the interaction between adaptation to oxidative stress and cell death is also important for understanding the nature of redox biology and disease pathogenesis.

## Materials and methods

### The fungus *Conidiobolus coronatus*

*C*. *coronatus* (Entomopthorales), isolate number 3491, originally isolated from *Dendrolaelaps* spp., was received from the collection of Prof. Bałazy (Polish Academy of Sciences, Research Center for Agricultural and Forest Environment, Poznań).

The fungal colonies were routinely cultured in 90 mm Petri dishes on Sabouraud agar medium. They were incubated at 20˚C under a 12-hour photoperiod (L:D 12:12) to stimulate sporulation [62]. Homogenized *G*. *mellonella* larvae were added to the medium to a final concentration of 10% wet weight (SAB-GM) in order to elevate virulence. The fungal colonies used for the experiments were cultured for seven days.

### Insects

Wax moths, *G*. *mellonella* (Pyralidae, Lepidoptera), were reared in glass chambers at 30°C, 70% relative humidity and in constant darkness on a semi-artificial diet prepared as described by Sehnal (1966) [63]. The larvae used in the experiments were five-day-old last instar larvae (5DL7) that had just reached their final (seventh) instar, having ceased feeding before entering metamorphosis. The 5DL7 larvae were exposed for 24 hours to fully-grown and sporulating *C*. *coronatus* colonies (24 hpi–hours post infection group). After these 24 hours of exposure, some of the insects were transferred to new, clean Petri dishes for another 24 hours (48 hpi group) with appropriate food. Around 15 individuals were maintained in each Petri dish, and a control group was formed of larvae exposed for 24 hours to sterile SAB-GM medium. This was found to be the most efficient method of infection, being the one that most closely resembles the natural infection process [64]. For each group, three independent replicates were performed.

### Hemolymph collection

The larvae were first washed with distilled water and then immersed briefly in 70% (v/v) ethanol to sterilize their surfaces and reduce contamination. Larval hemolymph was then collected from incisions in the last proleg. Freely-dripping hemolymph was collected into sterile polypropylene 1.5 ml centrifuge tubes preloaded with Grace Insect Medium (GIM; Gibco) with added gentamycin (10 mg/ml; Gibco), amphotericin B (250 μg/ml; Gibco) and 0.1 mM phenylthiourea (PTU; Sigma-Aldrich). Fresh hemolymph (8–10 drops; one drop 26 μl contains about 1.3 x 10^5^ of cells) was mixed with 300 μl of GIM. The hemolymph suspension was immediately transferred to μ-slide VI cell culture plates (IBIDI). Each plate hole was filled with 100 μl of hemolymph mixed with GIM. Following this, the plates were incubated for four or 24 hours, depending on the group, at 27°C and 80–90% humidity.

### Hemocyte staining

For cytoskeleton visualization, a fresh mix of hemolymph was incubated for 24 hours on IBIDI cell culture plates; following this, the hemocytes were fixed in 4% paraformaldehyde (PFA) (Sigma-Aldrich) in phosphate-buffered saline (PBS) (Sigma-Aldrich), and incubated with 1% BSA / 10% normal goat serum / 0.3M glycine in 0.1% PBS-Triton X-100 (Sigma-Aldrich) to permeabilize them and block non-specific protein-protein interactions. ActinGreen 488 Ready Probes Reagent (Invitrogen) was used to label the actin fibers. The cell nuclei were stained with DAPI (Enzo Life Sciences). The obtained samples were analyzed using an AxioVert A1 fluorescence microscope (Zeiss) with Zen software (Zeiss).

A very similar process to that used for cytoskeleton staining was used for hemolymph collection and the preparation for autophagy determination; the only difference was the time that the hemolymph was incubated on the plate. To visualize the autophagosomes, fresh hemolymph was mixed with 300 μl of GIM. Each plate hole was filled with 100 μl of hemolymph mixed with GIM. Plates were incubated for four hours at 27°C and 80–90% humidity. Following this, each sample was covered with 100 μl of Dual Detection Reagent (CYTO-ID® Autophagy detection kit—Enzo Life Sciences) and the cells were incubated in darkness for 30 minutes at 37°C. The cells were then washed and fixed in 4% PFA- PBS solution and the cell nuclei were stained with DAPI.

For LC3 protein detection, plates with fresh hemolymph were incubated for four hours. Following this, the cells were fixed in 4% PFA- PBS and incubated with 1% BSA / 10% normal goat serum / 0.3M glycine in 0.1% PBS-Triton X-100. To LC3 protein staining in hemocytes, LC3 monoclonal antibody (fluorescein labeled; concentration 1:100) (Enzo Life Sciences) was used. ActinRed 555 Ready Probes Reagent (Invitrogen) was used to label the actin fibers and DAPI for the cell nuclei. The hemocytes were observed under an AxioVert A1 fluorescence microscope (Zeiss) with Zen software (Zeiss).

### Evaluating the oxidative stress processes taking place in larval hemolymph and measuring the levels of autophagy proteins

The control and infected larvae were placed on ice to anaesthetize them. The hemolymph collected from 15 larvae was mixed with 100 μl of insect physiological saline (IPS) with PTU (0.1 mM) in order to inhibit melanization. Processes associated with oxidative stress were measured using ALDetect™ Lipid Peroxidation Assay Kit, Catalase Fluorometric Detection Kit, Cu/Zn-Superoxide Dismutase ELISA kit, DNA Damage ELISA Kit and Glutathione Peroxidase Assay Kit (all kits were from Enzo Life Sciences). To measure the concentration of autophagy proteins, the NBR1 ELISA and p62 ELISA kits (Enzo Life Sciences) were used. Samples were prepared according to the instructions given in the ELISA kits. Each test was performed in three independent replicates.

### Determination of total protein concentration

During preparation of the Glutathione Peroxidase Assay Kit, total protein concentration in the hemolymph of all samples was determined using a Bio-Rad Protein Assay kit (Bio-Rad Laboratories, Inc., Hercules, CA, USA). Bovine serum albumin (BSA) was used as the reference standard.

## Statistics

The obtained results were tested using the nonparametric Student’s t-test, with the results being significant at p≤0.05. Data are given as means ± standard deviations. GraphPad Prism 7 software was used for statistical calculations. The obtained p-values are presented in the Results section.
